# Can detomidine replace medetomidine for pharmacological semen collection in domestic cats?

**DOI:** 10.1590/1984-3143-AR2021-0017

**Published:** 2021-07-02

**Authors:** Maitê Cardoso Coelho da Silva, Karitha Marques Ullony, Gediendson Ribeiro de Araújo, Pedro Nacib Jorge-Neto, Verônica Batista Albuquerque, Simone Marques Caramalac, Alice Rodrigues de Oliveira, Ricardo Zanella, Mariana Groke Marques, Antonio Carlos Csemark, Thiago Cavalheri Luczinski, Fabrício de Oliveira Frazílio, Eliane Vianna da Costa e Silva, Thyara de Deco-Souza

**Affiliations:** 1 Faculdade de Medicina Veterinária e Zootecnia, Universidade Federal de Mato Grosso do Sul, Campo Grande, MS, Brasil; 2 Instituto Reprocon, Campo Grande, MS, Brasil; 3 Instituto de Biociências, Universidade Federal de Mato Grosso do Sul, Campo Grande, MS, Br.asil; 4 Faculdade de Medicina Veterinária e Zootecnia, Universidade de São Paulo, São Paulo, SP, Brasil; 5 Faculdade de Agronomia e Medicina Veterinária, Curso de Medicina Veterinária, Universidade de Passo Fundo, Passo Fundo, RS, Brasil; 6 Embrapa Suínos e Aves, Concórdia, SC, Brasil

**Keywords:** urethral catheterization, α_2_-adrenoceptor agonist, felids, tomcats

## Abstract

Among the different methods used for semen collection from domestic cats, the pharmacological collection by urethral catheterization becomes disruptive. Medetomidine is the elected α^2^-adrenoceptor agonist for that, but in several countries, it is not commercially available. This study aimed to evaluate the efficacy of detomidine compared to medetomidine in collecting semen by urethral catheterization in domestic cats. Urethral catheterization was performed on 13 mongrel cats using a disposable semi-rigid tomcat urinary catheter. Of the 19 semen collections performed with medetomidine induction, 94.7% were successful, while with detomidine induction, only 56.3% of 16 were successful. The values semen samples variables were as follows for volume - 10.56 ± 0.4 vs 8.88 ± 0.5 mL, motility - 171.67 ± 0.79 vs 49.77 ± 3.45%, vigor – 4.1 ± 0.03 vs 3.10 ± 0.1 and concentration - 3.24 ± 0.19 vs 2.15 ± 0.13 ×10^9^ sperm/mL respectively for medetomidine and detomidine group. The failure in semen collections with detomidine was mainly due to azoospermic samples, poor urethral relaxation, insufficient volume, or contamination of urine. The sperm concentration was also lower in the detomidine group (P <0.05) when compared to medetomidine. However, when the volume of semen collected was compared, we found no statistical differences. Despite its low performance in collecting semen from cats, detomidine may be an alternative when medetomidine is not accessible.

## Introduction

Interest in sperm collection and evaluation has grown significantly over the past decade in domestic cats. The use of domestic cats is undoubtedly an important model for the development of reproductive biotechnologies for wild felids (Jorge-Neto et al., 2020a). Electroejaculation was the method of choice for feline semen collection, resulting in a greater-volume lower-concentration sample and may have urinary and bacterial contamination (Zambelli et al., 2008). The pharmacological semen collection by urethral catheterization – using an α_2_-adrenoceptor agonist (α2A) – was described by Zambelli et al. (2008) and became a disruptive method for obtaining semen from felids, resulting in a lower-volume greater-concentration sample.

The α2A leads to smooth muscle contraction of the *vas deferens* in felids, promoting the semen release in the urethra. This allows the semen recovery by urethral catheterization, without urine contamination (Zambelli et al., 2008). In terms of the pharmacological profile of α2A agents, medetomidine is the most selective compound, with a relative α_1_/α_2_ selectivity ratio of 6.2 times more than detomidine (1.620 vs 260). Due to this, medetomidine is ﻿considered superior to others α2A and is known as a potent, selective, and specific agonist (Virtanen et al., 1988).

However, even with the medetomidine superiority for semen collection, in several countries it is not commercially available, making it impossible to be used in felids. To qualify another α2A agent for pharmacological semen collection, we compared detomidine with medetomidine for semen collection efficacy using domestic cats as a model for wild cats.

## Material and methods

This experiment was conducted at the multipurpose laboratory at the Faculty of Veterinary Medicine and Animal Science of Federal University of Mato Grosso do Sul (FAMEZ/UFMS; Campo Grande, MS, Brazil, 20°30'32.0”S 54°37'15.1”W). The present study was approved by the Ethics Committee on Animal Use of UFMS (#726/2015). The cat owners signed a consent form containing all the information regarding the experiment before each procedure.

### Animals and design

Thirteen healthy mongrel male cats were used, aged between one and six years old, weighing between 2.2 and 5.6 kg, and all presenting penile spines. Five animals were kept at the university’s cattery while seven were kept at their tutors’ house – some with street access. All the animals were fed with commercial cat food (different brands) and had access to water *ad libitum.* Males were fasted for 4 hours for water and 8 hours for food before the procedures.

Male cats were randomly assigned into two distinct protocols with minimal intervals of one week between semen collections: DETO (n = 16), were the association of detomidine (0.25 mg/kg; im) and ketamine (5 mg/kg; im) was administrated (Grove and Ramsay, 2000); and MEDE (n = 19), were medetomidine (0.1 mg/kg; im) and ketamine (5 mg/kg; im) was used (Araujo et al., 2020a). After all semen collection procedures were conducted, anesthesia was reversed using yohimbine (0.2 mg/kg; im). Semen collections were performed between 2015 and 2017 in April (n = 1), May (n = 1), June (n = 3), July (n = 4), August (n = 5), September (n = 1), October (n = 4) and November (n = 15).

### Semen collection

Semen collection was performed by urethral catheterization adapted from Araujo et al. (2018). Briefly, ~20 min after DETO or METE protocol administration, a disposable semi-rigid tomcat urinary catheter (w/ open end, 3FR, 130mm long) was 7−9 cm introduced carefully into the urethra. A 1-mL syringe was connected to the catheter, and negative pressure was applied to increase the suction effect. In those animals in which the semen was not collected in the first catheterization, transrectal prostatic massage was performed with the index finger, and then a new attempt was made (Araujo et al., 2018; Zambelli et al., 2008). Each semen collection attempt lasted from 30 to 60 seconds of urethral catheterization. Collection failure was considered when there was no urethral relaxation, making tomcat insertion difficult; urine contamination; azoospermia (< 1 x 10^6^ sperm/mL); and low recovered volume (< 2 µL).

### Semen analysis

Immediately after collection, semen samples were diluted into 40 µL pre-warmed at 37 °C egg yolk-free semen extender (CaniPlus Chill LT, Minitübe, Germany) and subjected to conventional sperm analysis such as volume (mL); motility (%) and vigor (wave motion scoring system, from 0 to 5; Evans & Maxwell, 1987). Volume was measured with an adjustable volume pipette discounting the pre-added extender volume (40 µL). A 2 µL drop of semen was placed on a prewarmed slide, covered with a coverslip, and examined using a bright-field microscope (magnification ×100; Axiostar – Carl Zeiss, Germany) with a heated stage at 37 °C for motility and vigor evaluation. Concentration measurements were made in a Neubauer chamber.

### Statistical analyses

All data were analyzed using the R-statistical program and MedCalc (Versão 19.5.3). Initially, the Fisher Exact test was used to verify the success rate among the different treatments. The data normality was affeered based on the Shapiro-Wilk normality test. Since none of the evaluated parameters followed a normal distribution (P < 0.05), the Kruskal Wallis test was used to compare medians among the groups, only in the samples with success in the sperm collection. Significance was considered if P < 0.05.

## Results

**Figure 1:**
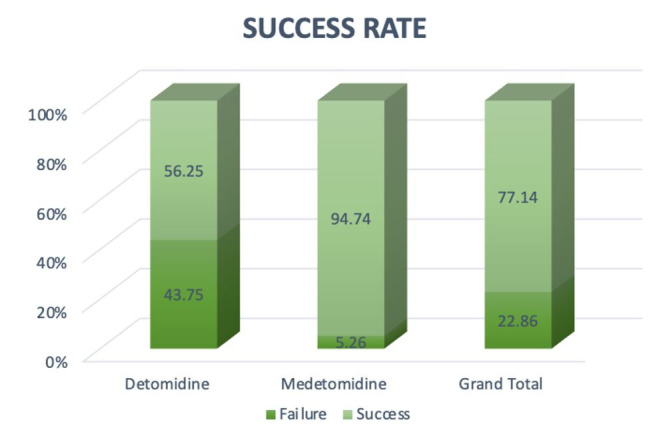
Graphical representation of success and failure rates of pharmacological semen collection in cats using medetomidine (*n*=19) and detomidine (*n*=16).

Both protocols allowed to recover semen samples from the tomcats. The success rate was 94.7% (18 out of 19) for MEDE and 56.3% (9 out of 16) for DETO (P = 0.012; Fig 1). Urinary contamination (n = 1) occurred in the MEDE group. In the DETO group, the collection failures were azoospermia (n = 4), poor urethral relaxation (n =1), insufficient volume (n =1) and urinary contamination (n = 1). Else for the DETO group, three ejaculates from different males were oligospermic (<1×10 ^6^ total sperm). Although failure occurred in only one collection due to the lack of urethral relaxation, all animals in the DETO group presented urethral resistance during catheterization.

In addition to the lower efficiency of the semen collection procedures, the use of detomidine also resulted in less concentrated samples (P = 0.019) with lower vigor (P = 0.001) and motility (P = 0.006). Only the ejaculated volume (P=0.53) presented no significant differences (Table 1).

**Table 1:** Semen variables assessed by urethral catheterization after induction using detomidine (*n*=9) or medetomidine (*n*=18) in cats.

	Medetomidine	Detomidine
Volume (µL)	10.56 ± 0.4^A^	8.88 ± 0.5^A^
Vigor	4.11 ± 0.03^A^	3.10 ± 0.1^B^
Motility (%)	71.67 ± 0.79^A^	49.77 ± 3.45^B^
Concentration (sperms × 10^9^/mL)	3.24 ± 0.19^A^	2.15 ± 0.13^B^
Total Concentration (× 10^9^ sperms)	21.68 ± 1.2^A^	12.77 ± 1.89^B^

Data represented as the mean ± standard error. Values with different letters on the same line differed from each other (*P* <0.05).

The results of multiple collections of the same animal presented statistical differences between them, which can be explained by environmental and individual factors of the animals. However, when semen collections based on treatment were compared, we did not find statistical differences, demonstrating that despite the differences between the different time-points of semen collections, the dependent variables for each treatment did not show any effect (Table 2).

**Table 2:** Evaluation of the dependent variables regarding collection, treatment, and collection × treatment ratio (*P* <0.05).

	**R^2^**	***P*-mod**	**CV**	**Collect**	**Treatment**	**Collection × treatment**
Volume	0,2012	0.3472	86.03	0.34	0.31	0.63
Concentration*	0.5558	0.0070	93.13	0.10	0.0010	0.45
Total concentration**	0.5169	0.0025	95.09	0.26	0.0269	0.46
Vigor	0.6265	<0.0001	42.63	0.0120	0.0001	0.51
Motility	0.6434	<0.0001	51.03	0.0345	<0.0001	0.69

* Results following the removal of the out layer >3000. **Results following the removal of the out layer >50.

## Discussion

Very limited information on the use of detomidine in felines is available. To the best of our knowledge, the present study was the first in which detomidine was used for feline semen collection thru urethral catheterization. Medetomidine, however, is widely used for this purpose as it is known to promote the release of sperm into the urethra, allowing the semen to be collected by urethral catheterization (Araujo et al., 2020b; Araujo et al., 2018; Jorge-Neto et al., 2020b; Jorge Neto et al., 2019; Lueders et al., 2012; Prochowska et al., 2015; Silva et al., 2019; Silvatti et al., 2020; Zambelli et al., 2008). Thereby, this study aimed to assess whether detomidine had the same effect as medetomidine for semen collection in domestic cats. In this study, 13 mongrel cats were used for pharmacological semen collection. Five were from the experimental cattery university and the others were obtained from owners with permission. The minimum interval of one week between the two collections was intended to ensure sufficient time for sperm replacement.

In combination with ketamine, the intramuscular doses of detomidine are 0.05 to 0.06 mg/kg for lions (Fyumagwa et al., 2012; Kolata, 2002) and 0.15 mg/kg in cougars (Albuquerque et al., 2016). In domestic cats, the dose used ranges from 0.1 mg/kg (Emiliano and Souza, 2017) up to 0.5 mg/kg (Grove and Ramsay, 2000; Wetzel and Ramsay, 1998). Due to the lower doses found in the literature, it was decided to use the lowest dose (0.25 mg/kg) administered intramuscularly used by Grove & Ramsay (2000). This decision was made aiming at the safety of the animals during the procedure, avoiding side effects during the administration of α2A agents, such as sinus bradycardia and heart murmur (Grove and Ramsay, 2000; Luczinski et al., 2020).

Both protocols allowed to collect semen in cats, however, the success rates were higher with medetomidine (94.7%) compared to detomidine (56.3%). Medetomidine, dexmedetomidine, and detomidine are known to be α2A sedatives with greater binding specificity for α_2_-adrenergic receptors (R-α-2). However, they are not specific only to these receptors but also act on the α_1_-adrenergic receptors (R-α-1). Nevertheless, medetomidine and dexmedetomidine are more specific than detomidine (1620:1, 1620:1, and 260:1 α_2_:α_1_-receptor binding specificity ratios, respectively) (Maddison et al., 2008; Muir and Hubbell, 2014; Virtanen et al., 1988). Thus, a higher success rate was expected for medetomidine.

There was urethral resistance during urethral catheterization after detomidine induction in all animals. R-α-2 are located at the central and peripheral levels in the pre- and post-synaptic membranes. In the urogenital tract, stimulation of these receptors promotes relaxation of the *vas deferens* and post-prostate urethral muscles (Maddison et al., 2008; Virtanen et al., 1988). Therefore, it is likely that the lower selectivity of detomidine for R-α-2 binding resulted in a lesser ability to relax the vas deferens and the urethra, resulting in greater sensitivity to urethral catheterization and less release of sperm into the urethra. Among the causes of failure in the efficiency in detomidine induction (azoospermia, insufficient urethral relaxation, insufficient semen volume, and urine contamination), three animals submitted to the detomidine protocol were considered oligospermic. The difficulty of urethral catheterization in this group may have contributed to oligospermia.

There was less efficiency in semen collection after detomidine induction, and most males had less concentrated sperm with low vigor and motility (P <0.05; Table 1). These differences may be related to the specificity of R-α-2 binding of each sedative. The detomidine had underperformed the medetomidine for presenting lower specificity, causing insufficient relaxation of the *vas deferens* and thus resulting in a shortage of sperm into the urethra. The lower sperm concentration found in the detomidine group compared to medetomidine resulted in a greater amount of seminal fluid. This can lead to osmotic changes, which negatively affect the sperm membrane and interfere with sperm viability, as reported by (Sousa et al., 2016) in six-banded armadillos. The only parameter that did not show any statistical difference was the semen volume.

All animals submitted to more than one semen collection procedure following the same treatment were kept in the experimental cattery after the first semen collection. Stress can lead to poor sperm production both quantitatively and qualitatively (Axnér, 2006; Dobson et al., 2001; Dobson and Smith, 2000). These animals showed better sperm quality in the first collection than the following ones, possibly due to the stress of adapting to the new environment.

Different doses of medetomidine were compared by Cunto et al. (2015) for pharmacological collection of semen in cats. It was observed that the semen collected with the lowest dose (50 µg / kg) of medetomidine showed lower concentration, volume, vigor, and motility in relation to that collected with the highest dose (130 µg / kg). Even more, the lower dose resulted in less sedation and less urethral relaxation, making urethral catheterization harder. These results corroborate our results, in the pharmacological semen collection method, showing that weaker stimulation of R-α-2 leads to less efficiency in the collection process and lower semen quality.

Further studies are needed to assess the appropriateness of the dose of detomidine for the pharmacological semen collection. The dose adequacy should also consider the relative α_1_/α_2_ selectivity ratio. Only increasing the dose aiming to approach the selectivity of medetomidine (6.2x higher than detomidine) will also activate the R-α-1, which may compromise the result. Even more, it may bring collateral effects that may be irreversible to the animal.

## 5. Conclusions

Detomidine can be used as a substitute for medetomidine for the pharmacological semen collection in cats. However, medetomidine should be the drug of choice when available due to its superior performance in successful sperm collection.
